# Supplementary material for: Cryo-EM structure of single-layered nucleoprotein-RNA complex from Marburg virus
Source: Nat Commun. 2024 Nov 27;15:10307. doi: 10.1038/s41467-024-54431-7 (PMC11603049; doi:10.1038/s41467-024-54431-7)
Supplement: Supplementary file 1 — Supplementary Information [file 41467_2024_54431_MOESM1_ESM.pdf]

Cryo-EM structure of single-layered nucleoprotein-RNA complex from Marburg virus

Luca Zinzula [1,2] *, Florian Beck [1,3], Marianna Camasta [1], Stefan Bohn [1,4], Chuan Liu [1],
Dustin Morado [5,6], Andreas Bracher [7], Jürgen M. Plitzko [1,3], Wolfgang Baumeister [1,2] *

[1] Max Planck Institute of Biochemistry, Department of Molecular Structural Biology, 82152 Martinsried, Germany
[2] iHuman Institute, ShanghaiTech University, 201210 Shanghai, China.
[3] Max Planck Institute of Biochemistry, CryoEM Technology, 82152 Martinsried, Germany.
[4] Institute of Structural Biology, Helmholtz Center Munich, 85764 Oberschleissheim, Germany.
[5] Max Planck Institute of Biochemistry, Department of Cell and Virus Structure, 82152 Martinsried, Germany.
[6] Stockholm University, Department for Biochemistry and Biophysics, Science for Life Laboratory, 10691 Stockholm, Sweden.
[7] Max Planck Institute of Biochemistry, Department of Cellular Biochemistry, 82152 Martinsried, Germany.
* corresponding authors: zinzula@biochem.mpg.de ; baumeist@biochem.mpg.de

Supplementary Information

a

microscope	camera	pixel size (Å)	defocus range (µm)
300 kV Titan Krios G4	Falcon 4	0.93	-0.5 – 3.0
total dose	micrographs	autopicked particles	particles in final reconstruction
50 e- Å^{-2}	5,518	67,385	17,118

b

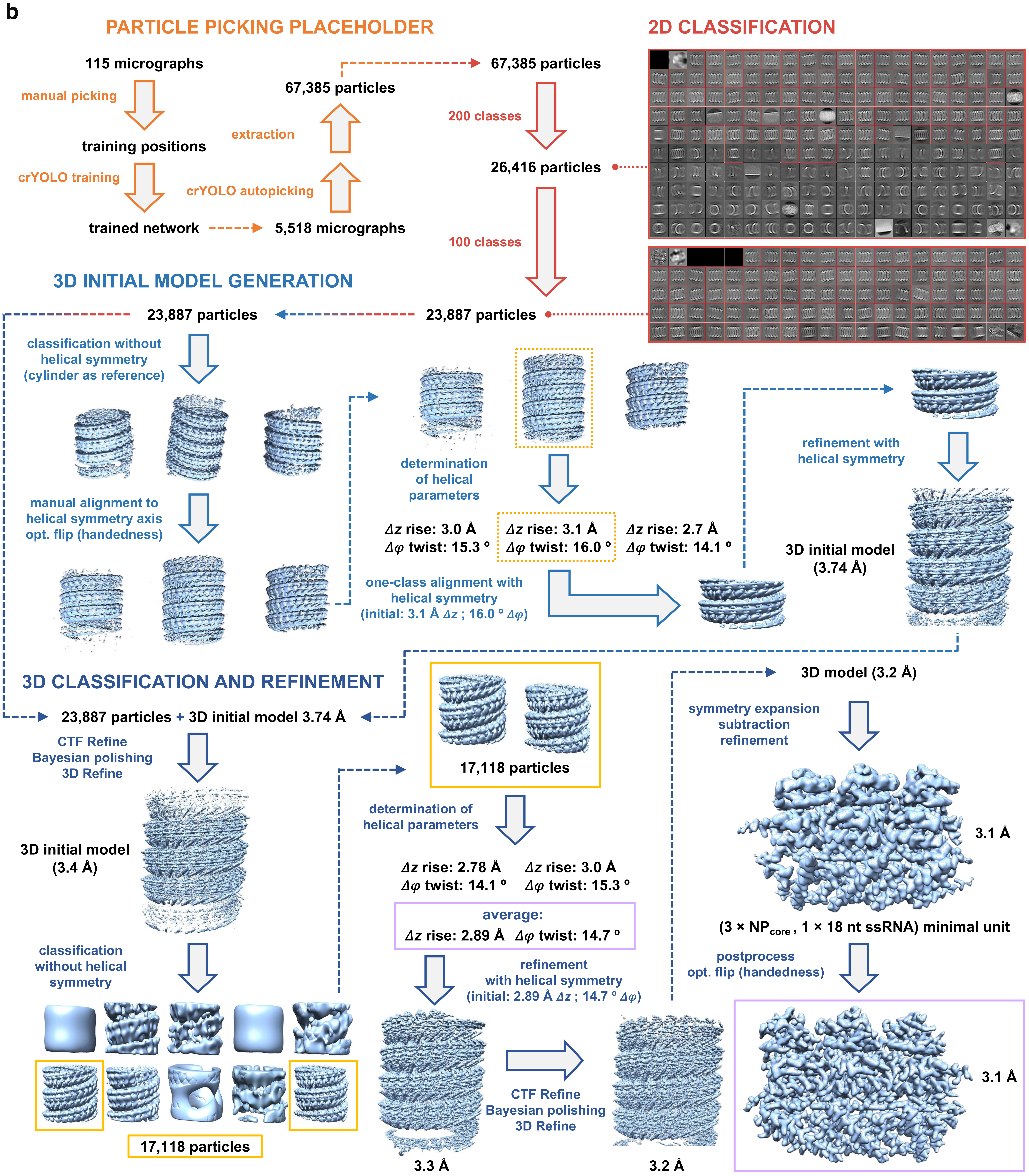

Supplementary Fig. 1. Experimental workflow for structure determination of MARV RNP complex by helical reconstruction. a, cryo-EM data collection parameters and dataset composition. **b**, flow chart of the SPA helical reconstruction pipeline for the structure determination of the *in vitro* reconstituted MARV RNP complex in single-layer conformation; 2D averages (with selected best classes highlighted by red squares) and cryo-EM density maps of the initial, 3D-class best selected and refined models (highlighted by yellow and violet squares, respectively) are shown.

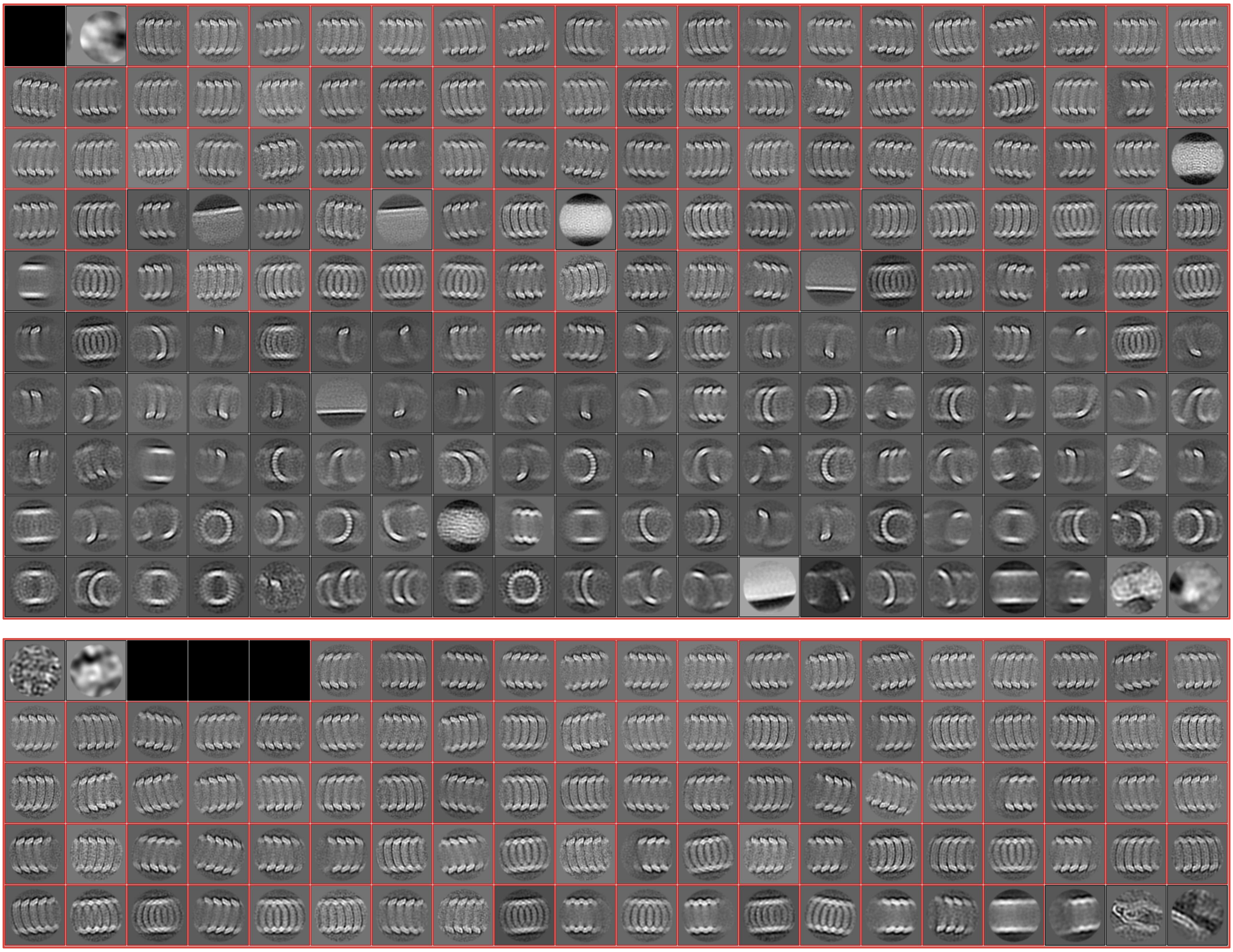

Supplementary Fig. 2. 2D classification of *in vitro* reconstituted MARV RNP complex cryo-EM images for structure determination by helical reconstruction. Zoomed view of the two rounds of 2D classification into 200 classes (first round, upper panel; starting dataset of 67,385 particles, final selected dataset 26,416 particles) and 100 classes (second round, lower panel; starting dataset 26,416 particles, final selected dataset 23,887 particles) shown in Supplementary Fig. 1, undertaken during the SPA workflow for the refinement of 2D class averages and the elimination of poorly aligned particles or contaminants; selected best classes at the end of each 2D classification step are highlighted by red squares.

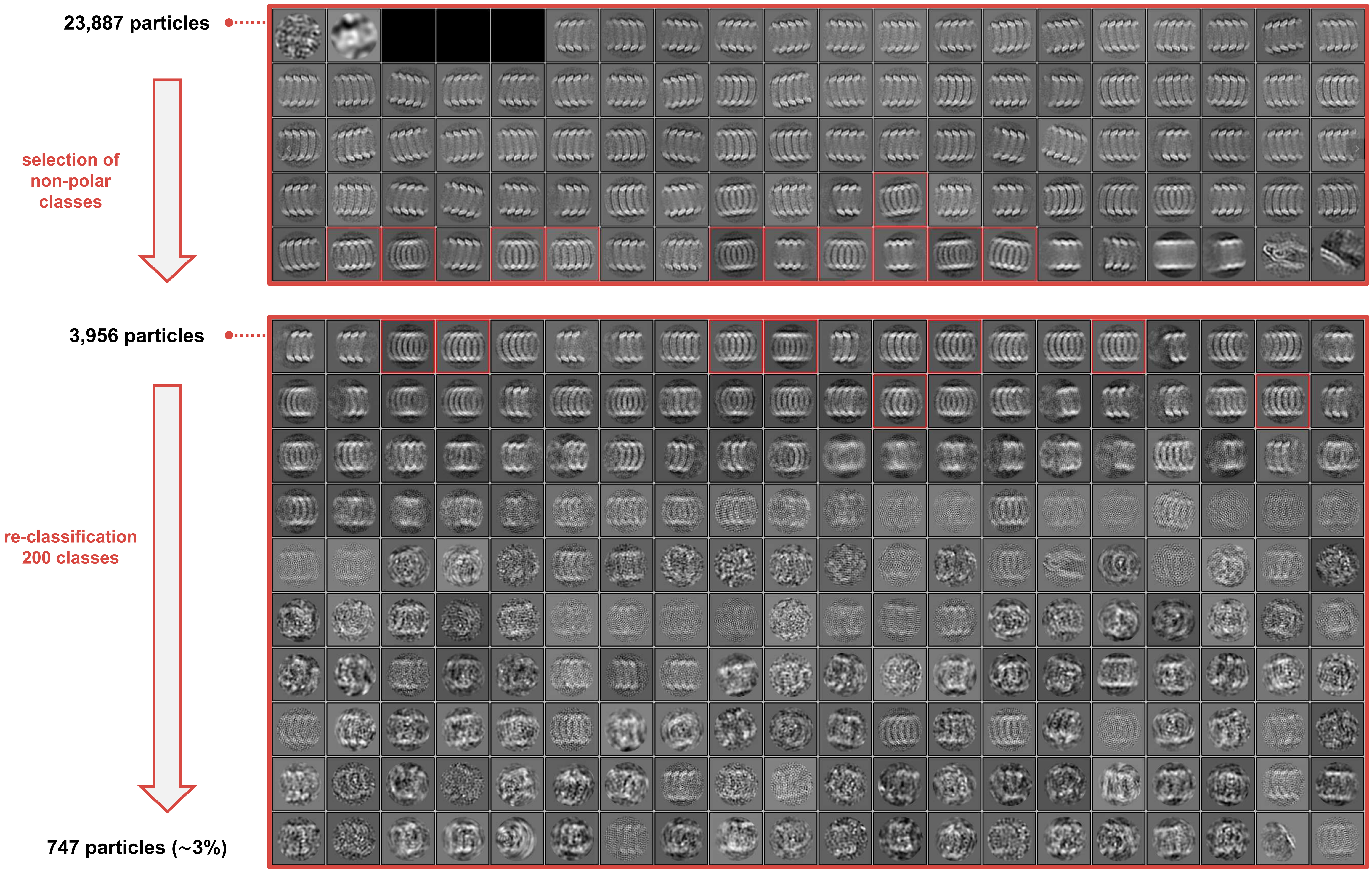

Supplementary Fig. 3. Assessment of structural heterogeneity in the cryo-EM dataset of the *in vitro* reconstituted MARV RNP complex. Selection of non-polar 2D class averages (n = 11; 3,956 particles) from the pool of the last round of 2D classification (23,887 particles), and re-classification over 200 classes for the new selection of particles whose 2D classes do not have any polarity in their helical course and display structural heterogeneity (n = 747; ~ 3 % of the dataset); selected best class averages at each step of 2D classification are highlighted by red squares.

Supplementary Table 1. Cryo-EM data collection, refinement and validation statistics

	Marburg virus single-layered nucleoprotein-RNA complex (EMDB-50803) (PDB 9FVD)
**Data collection and processing**	
Magnification	130,000 ×
Voltage (kV)	300
Electron exposure (e–/Å^2)	40
Defocus range (μm)	− 0.5 to − 3.0
Pixel size (Å)	0.93
Symmetry imposed	Helical
Initial particle images (no.)	67,385
Final particle images (no.)	17,118
Map resolution (Å)	3.2
FSC threshold	0.143
Map local resolution range (Å)	3.0 to 3.6
**Refinement**	
Initial model used (PDB code)	7F1M
Model resolution (Å)	3.2
Map sharpening *B* factor (Å^2)	88
Model composition	
Non-hydrogen atoms	10,146
Protein residues	1,143
Nucleotide residues	54
B factors (Å^2)	
Protein	115.06
Nucleotide	93.61
R.m.s. deviations	
Bond lengths (Å)	0.004
Bond angles (°)	0.945
Validation	
MolProbity score	1.83
Clashscore	16.91
Poor rotamers (%)	0
Ramachandran plot	
Favored (%)	97.51
Allowed (%)	2.49
Disallowed (%)	0

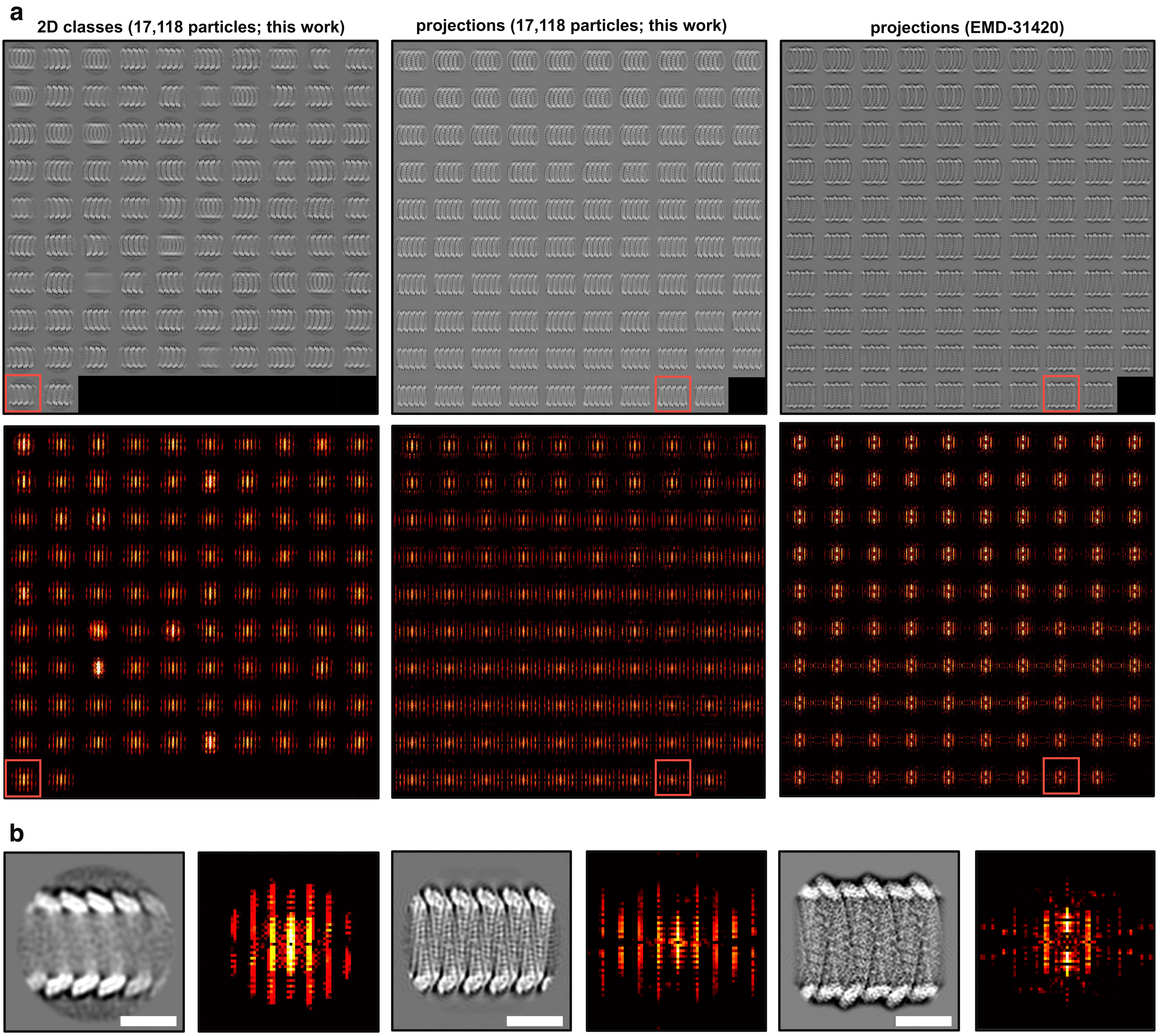

Supplementary Fig. 4. Power spectra analysis of the single- and double-layered MARV RNP complexes. Comparative overview of the class averages from 2D classification (upper left panel) and real space projections of the map (upper middle panel) of the single-layered MARV RNP complex (this work), with the real space projections of the map (upper right panel) of the double-layered one (EMD-31420), and their corresponding power spectra (left, middle and right lower panel, respectively); representative real space 2D class averages, projections of the map and power spectra, respectively, are highlighted by red squares. **b**, zoomed views of representative 2D class averages (left panel), real space projections of the map (middle panel) of the single-layered MARV RNP complex (this work), and real space projection of the map of the double-layered one (EMD-31420), with different pattern of layer lines shown by their respective power spectra (scale bar,15 nm).

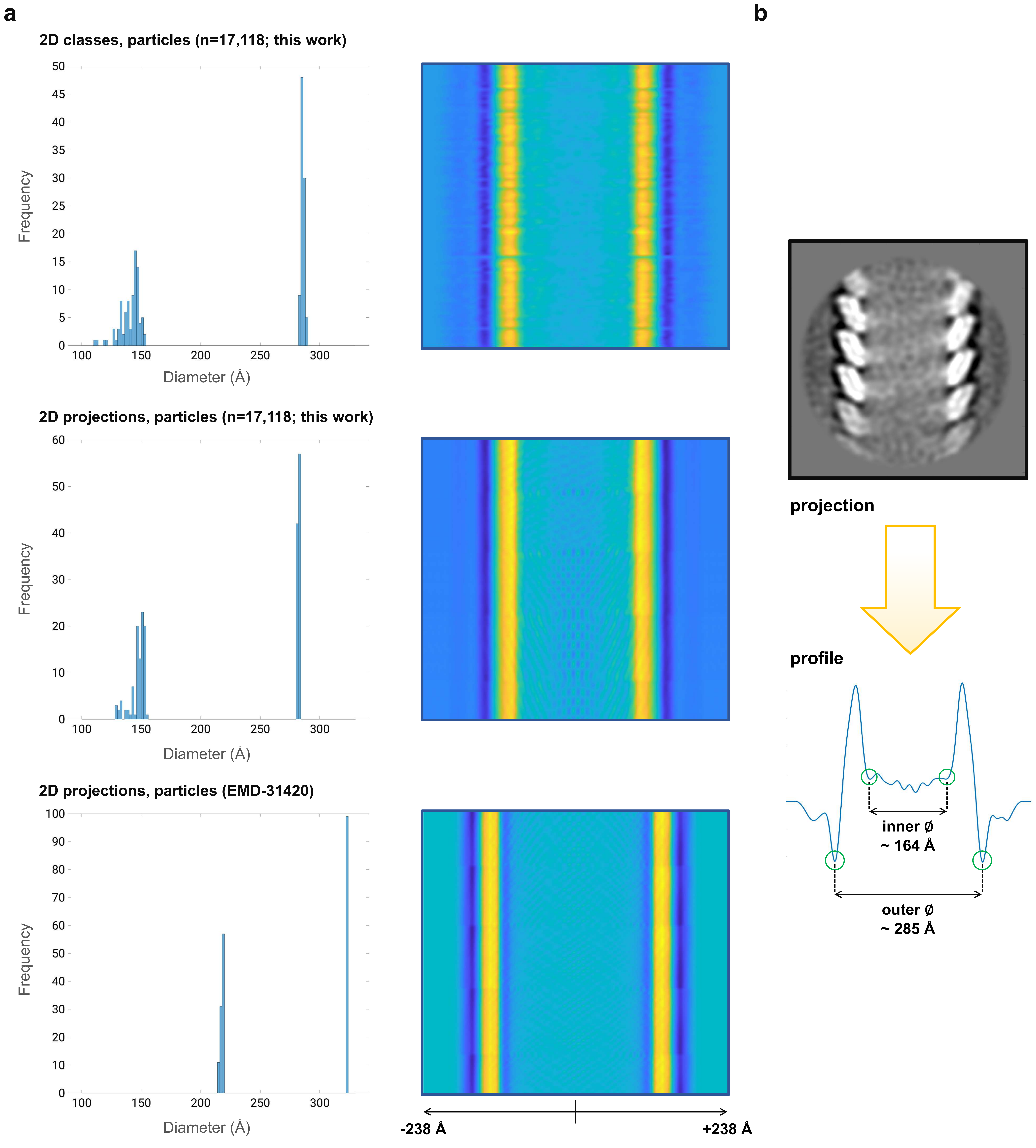

Supplementary Fig. 5. Diameter analysis of single- and double-layered MARV RNP complexes. a, comparative overview of histograms for inner and outer diameter (left panels) and stacked line profiles (right panels). **b**, representative determination of inner and outer diameter for one 2D-class average image.

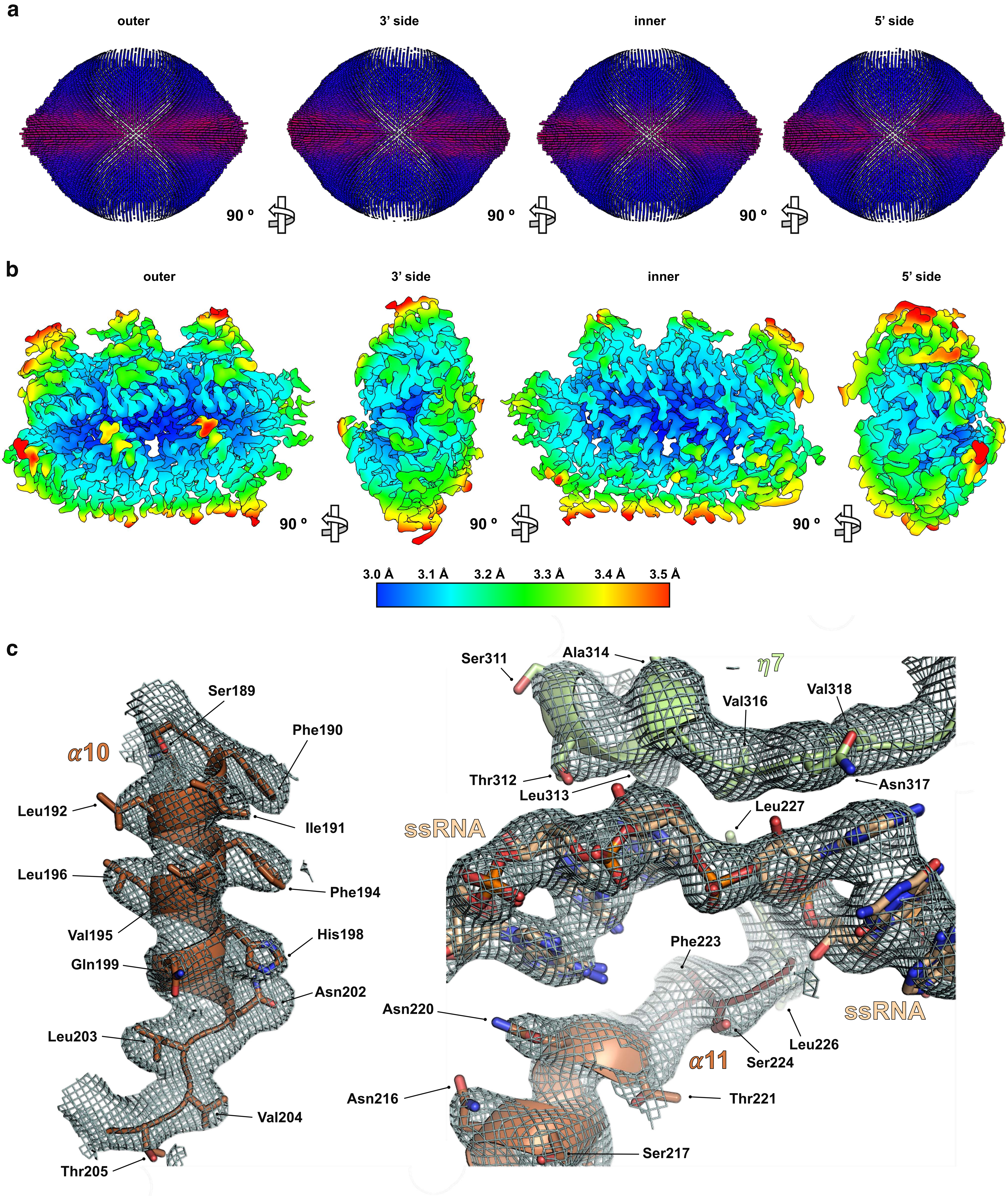

Supplementary Fig. 6. Cryo-EM analysis of the trimeric asymmetric unit of single-layered MARV RNP complex. a, Euler angle distribution of views that contributed to the 3D reconstruction of the single-layered MARV RNP complex trimeric repeat unit, shown in four orthogonal views. **b**, Local resolution of the cryo-EM density map of the single-layered MARV RNP complex trimeric repeat unit, shown in four orthogonal views (rainbow color scale bar, from 3.0 to 3.5 Å). **c**, Superposition of atomic model and cryo-EM density map of key structural elements of the single-layered MARV RNP complex protomer, including helix $\alpha10$ (left) and helices $\alpha11$, $\eta7$ and ssRNA (right); models and densities are shown in ribbon and mesh representation, respectively, with side chains of key residues fitted into the density map highlighted as sticks.

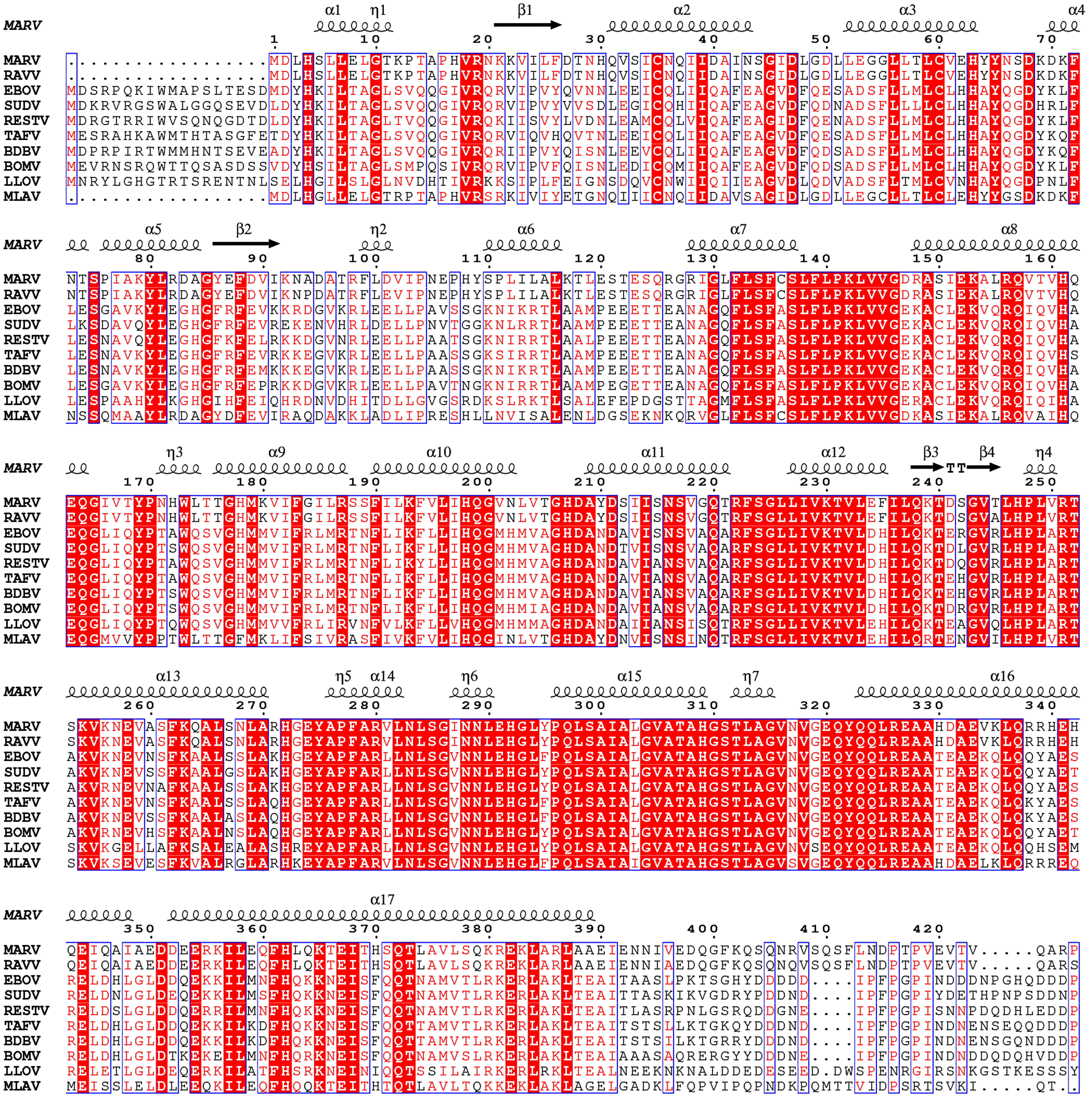

Supplementary Fig. 7. Comparative topology of filoviral NP_{core} based on MARV structure consensus. Multiple amino acid sequence alignment of reference NP sequences (aa 1-427, MARV numbering) within the family *Filoviridae*, performed using RefSeq amino acid sequences from NCBI Protein database YP_001531153.1 (MARV), YP_009055222.1 (RAVV), NP_066243.1 (EBOV), YP_138520.1 (SUDV), NP_690580.1 (RESTV), YP_003815423.1 (TAFV), YP_003815432.1 (BDBV), YP_009513274.1 (BOMV), YP_004928135.1 (LLOV) and YP_010087183.1 (MLAV). The secondary structure topology scheme from the MARV NP_{core} atomic model (this work) is used as consensus and displayed above the aligned sequences.

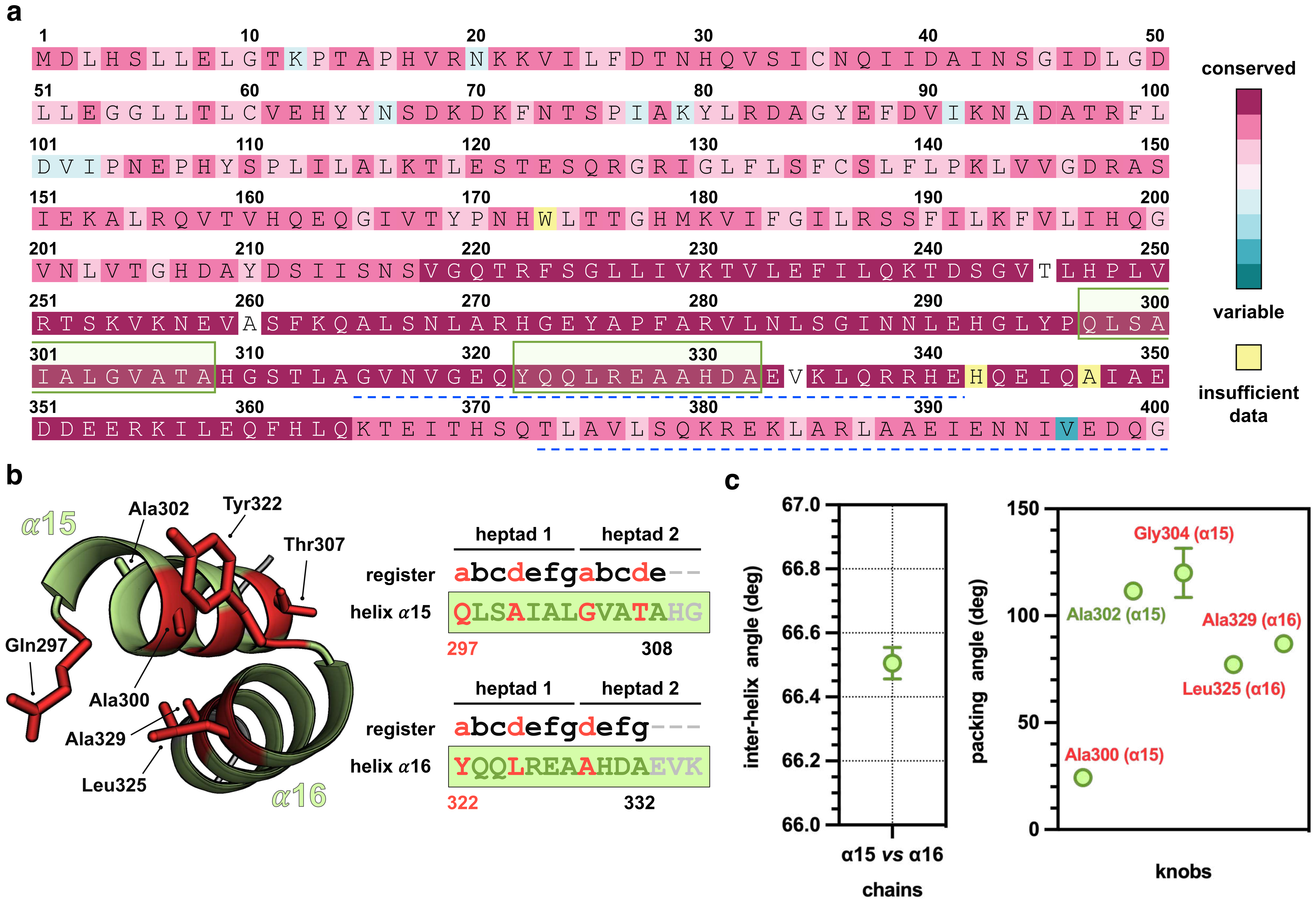

Supplementary Fig. 8. Highly conserved non-canonical coiled-coil motifs in MARV NP$_{core}$ C-lobe. **a**, amino acid residue similarity calculated within the *Orthomarburgvirus* genus over all 143 sequences available in the Bacterial and Viral Bioinformatics Resource Center (BV-BRC) database (https://www.bv-brc.org, last access on May 8th, 2023) and mapped onto the reference MARV NP sequence (aa 1-400, RefSeq NCBI Protein YP_001531153.1); 0% to 100% conservation is shown as teal-to-magenta scale, residue positions with insufficient data for conservation score calculation are shown in yellow; regions predicted to harbor a coiled-coil motif essential for NP oligomerization and viral RNA synthesis (as described in DiCarlo et al., 2007, *Virol J*) are highlighted by blue dashed underlines, whereas those within them identified in this work as involved in a dimeric intra-chain coiled-coil are boxed in green. **b**, ribbon representation (left panel) of the intra-chain non-canonical, dimeric coiled-coil established between C-lobe α15 and α16, with heptad register and per-chain amino acid sequence (right panel); coiled-coil knobs side chains and residue positions are highlighted in red, whereas residues that do not follow coiled-coil packing are highlighted in light gray. **c**, inter-helix (left) and knob-packing (right) angles in the intra-chain non-canonical dimeric coiled-coil of MARV NP$_{core}$ C-lobe; plotted values and errors are the mean and SD, respectively, measured among protomers of an arbitrary minimal hexameric unit within the RNP helix.

a

features	this work	PDB: 7F1M (Fujita-Fujiharu et al., 2022 *Nature Communications*)
	single-layered	double-layered
handness (*h*)	*h* (-), left-handed	*h* (-), left-handed
outer diameter	285 Å	330 Å
inner diameter	164 Å	255 Å
rise (*Δ*z)	2.87 Å	4.23 Å
twist (*Δφ*)	14.73 °	11.8052 °
n. subunits/turn	24.4	30.5
pitch (p)	70.14 Å	128.99 Å

b

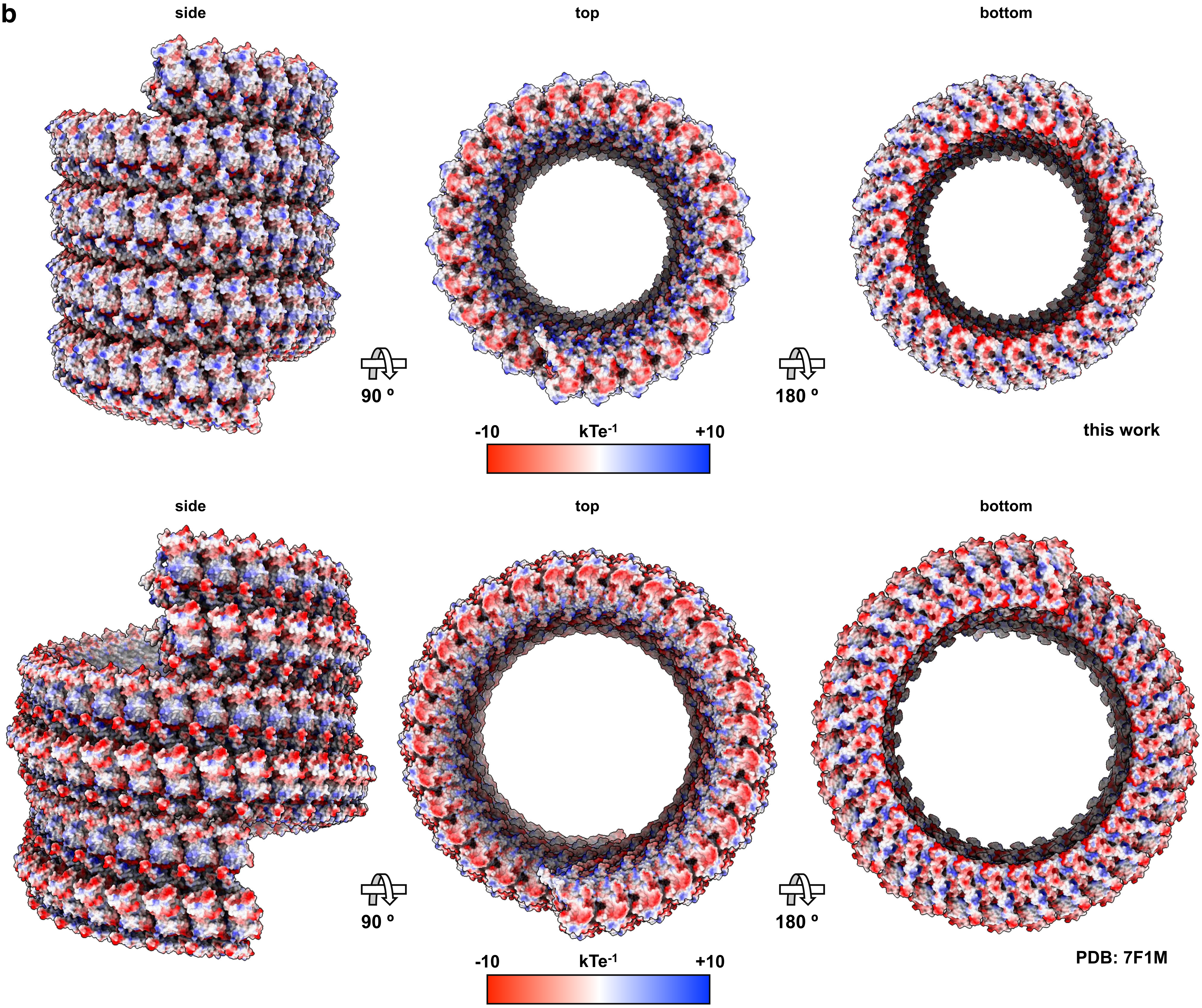

Supplementary Fig. 9. Different conformations of the MARV RNP complex. a, comparative table summarizing features and helical parameters that differ between the single-layered MARV RNP complex (this work) and the previously described double-layered one (PDB: 7F1M). **b**, electrostatic surface potential calculation illustrates differences in surface charge between the MARV RNP complex in single-layer (this work) and the one in double-layer (PDB: 7F1M) conformation, both displayed as isosurface representation in three orthogonal directions; red, white and blue regions represent areas of negative, neutral, and positive electrostatic surface potential, respectively (color scale bar, from -10.0 to +10.0 kT e-1).

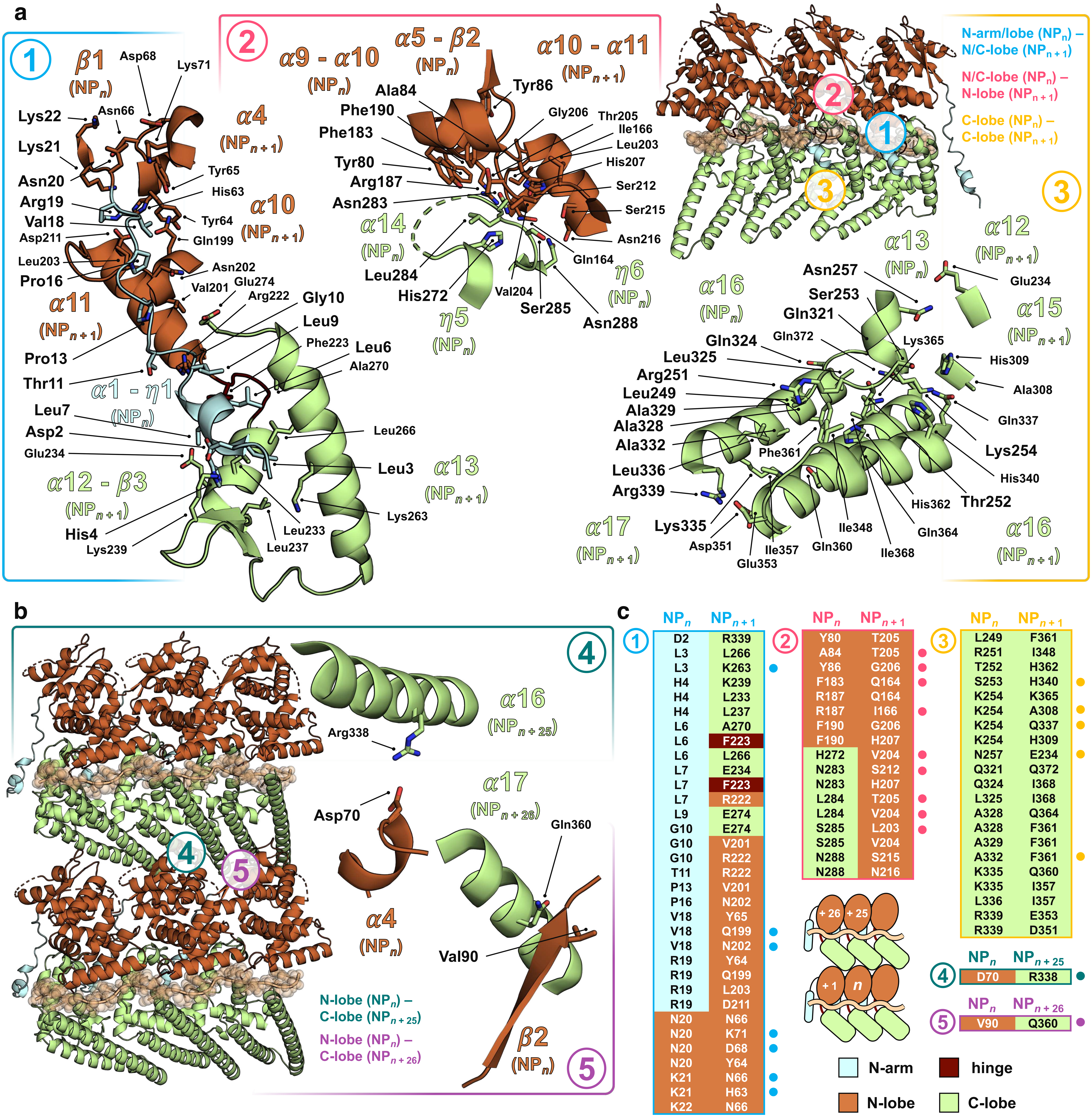

c ①

NP$_n$	NP$_{n+1}$	
D2	R339	
L3	L266	
L3	K263	•
H4	K239	
H4	L233	
H4	L237	
L6	A270	
L6	F223	
L6	L266	
L7	E234	
L7	F223	
L7	R222	
L9	E274	
G10	E274	
G10	V201	
G10	R222	
T11	R222	
P13	V201	
P16	N202	
V18	Y65	
V18	Q199	•
V18	N202	•
R19	Y64	
R19	Q199	
R19	L203	
R19	D211	
N20	N66	
N20	K71	•
N20	D68	•
N20	Y64	
K21	N66	•
K21	H63	•
K22	N66	

②

NP$_n$	NP$_{n+1}$	
Y80	T205	
A84	T205	•
Y86	G206	•
F183	Q164	•
R187	Q164	
R187	I166	•
F190	G206	
F190	H207	
H272	V204	•
N283	S212	•
N283	H207	
L284	T205	•
L284	V204	•
S285	L203	•
S285	V204	
N288	S215	
N288	N216	

③

NP$_n$	NP$_{n+1}$	
L249	F361	
R251	I348	
T252	H362	
S253	H340	•
K254	K365	
K254	A308	•
K254	Q337	•
K254	H309	
N257	E234	•
Q321	Q372	
Q324	I368	
L325	I368	
A328	Q364	
A328	F361	
A329	F361	
A332	F361	•
K335	Q360	
K335	I357	
L336	I357	
R339	E353	
R339	D351	

④

NP$_n$	NP$_{n+25}$	
D70	R338	•

⑤

NP$_n$	NP$_{n+26}$	
V90	Q360	•

Supplementary Fig. 10. Structural details of single-layered MARV RNP complex (NP$_{core}$ homo-oligomerization). **a**, ribbon representation of a trimeric minimal unit (top) and close-up (insets) showing the three types of intra-strand interactions between any arbitrary protomer NP$_n$ and its adjacent neighbor NP$_{n+1}$; interaction 1, involving the N-arm and N-lobe of NP$_n$, and the N-lobe, hinge and C-lobe of NP$_{n+1}$; interaction 2, involving the N-lobe and C-lobe of NP$_n$, and the N-lobe of NP$_{n+1}$; interaction 3, involving the C-lobe of NP$_n$ and the C-lobe of NP$_{n+1}$. **b**, ribbon representation of a hexameric minimal unit (left) and close-up (insets) showing the two types of inter-strand interactions between any arbitrary protomer NP$_n$ and its neighbors in the subsequent strand occupying the same position (NP$_{n+25}$) and the subsequent one (NP$_{n+26}$); interaction 4, involving the N-lobe of NP$_n$ and the C-lobe of NP$_{n+25}$; interaction 5, involving the N-lobe of NP$_n$ and the C-lobe of NP$_{n+26}$. **c**, summary of the interacting residues (within 4 Å distance) leading to the assembly of MARV NP$_{core}$ into the RNP complex via the five type of interactions between any arbitrary protomer NP$_n$ and its neighbors NP$_{n+1}$, NP$_{n+25}$ and NP$_{n+26}$; residue couples marked with a circle are unique (within 4 Å distance) of the MARV RNP complex in single-layer conformation.

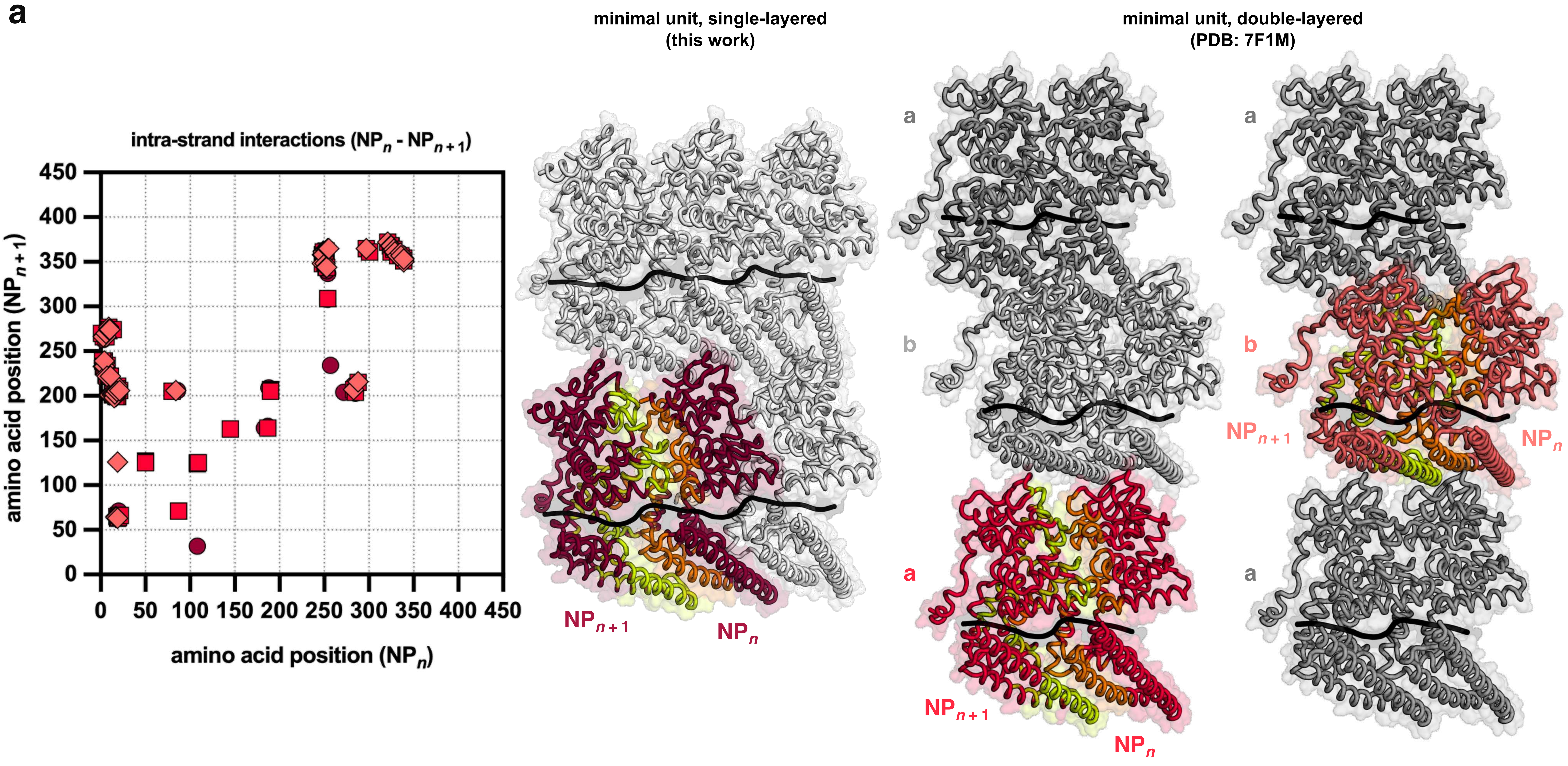

(4.0 Å distance cutoff)	number of interactions	residues from protomer NP_n	residues from protomer NP_{n+1}	interface area (Å^2)
NP (single-layered)	71	41	45	2593.5
NP-a (double layered)	73	43	46	2855.7
NP-b (double-layered)	84	35	41	2306.2

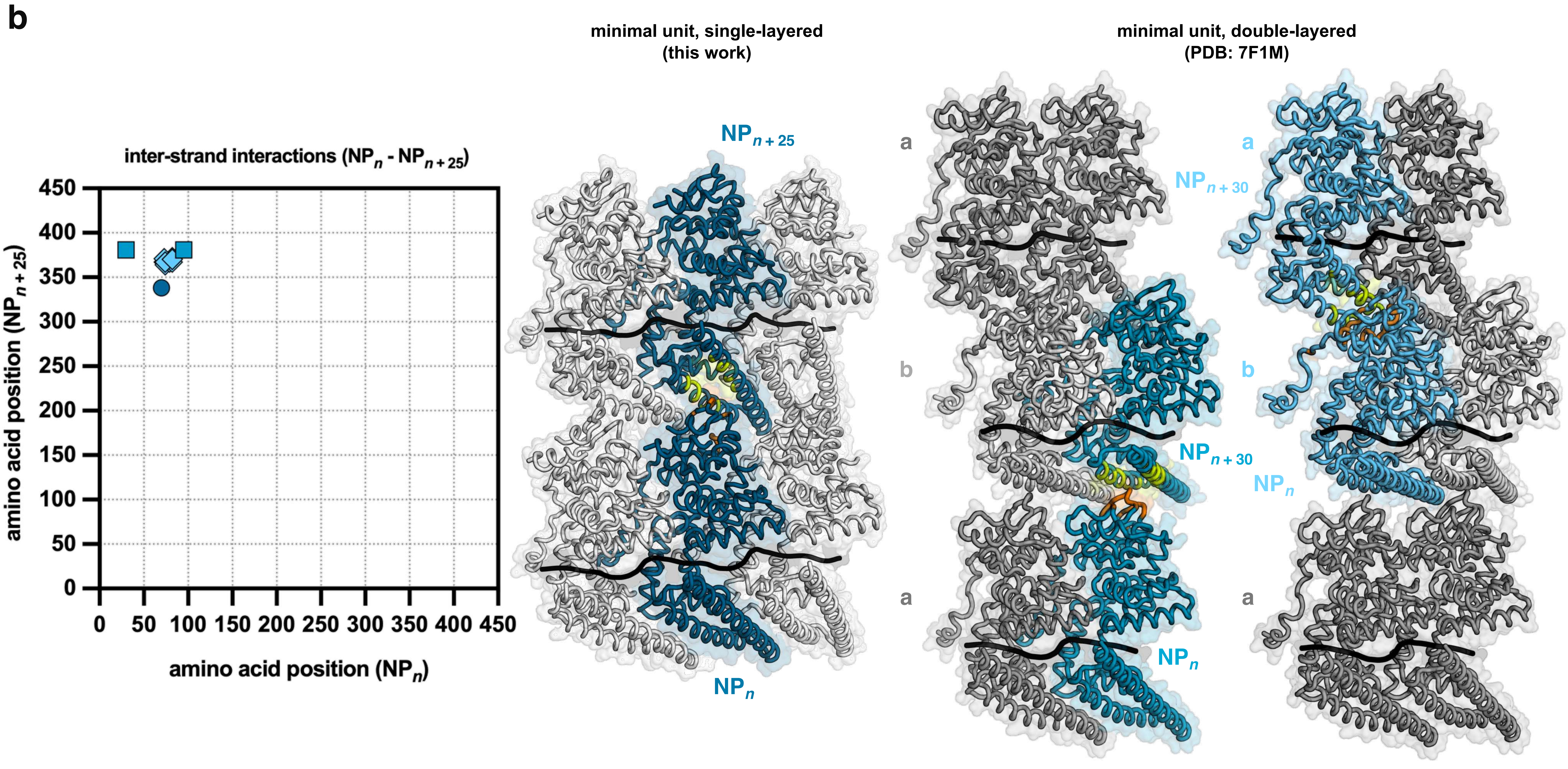

(4.0 Å distance cutoff)	number of interactions	residues from protomer NP_n	residues from protomer $NP_{n+25\,(30)}$	interface area (Å^2)
NP (single-layered)	1	1	1	61.2
NP-a (double layered)	2	2	1	237.2
NP-b (double-layered)	8	4	4	250.4

Supplementary Fig. 11. Structural analysis of NP_{core} - NP_{core} interactions in the two conformations of the MARV RNP complex. **a**, amino acid residue contacts map (left panel), ribbon/isosurface representation (right panel) and quantitative comparison (lower panel) of the intra-strand interactions between any given NP_n and its adjacent neighbor, NP_{n+1}. **b**, amino acid residue contacts map (left panel), ribbon/isosurface representation (right panel) and quantitative comparison (lower panel) of the inter-strand interactions between any given NP_n and the neighbor in the same position in the subsequent strand, NP_{n+25} (this work) or NP_{n+30} (PDB: 7F1M). For any arbitrary protomer NP in the single-, and NP-a and NP-b in the double-layer MARV RNP complex, respectively, the number of interactions, involved residues per protomer and extension of the interface area are indicated (circle, single-layered RNP NP; square, double-layered RNP NP-a; rhombus, double-layered RNP NP-b).

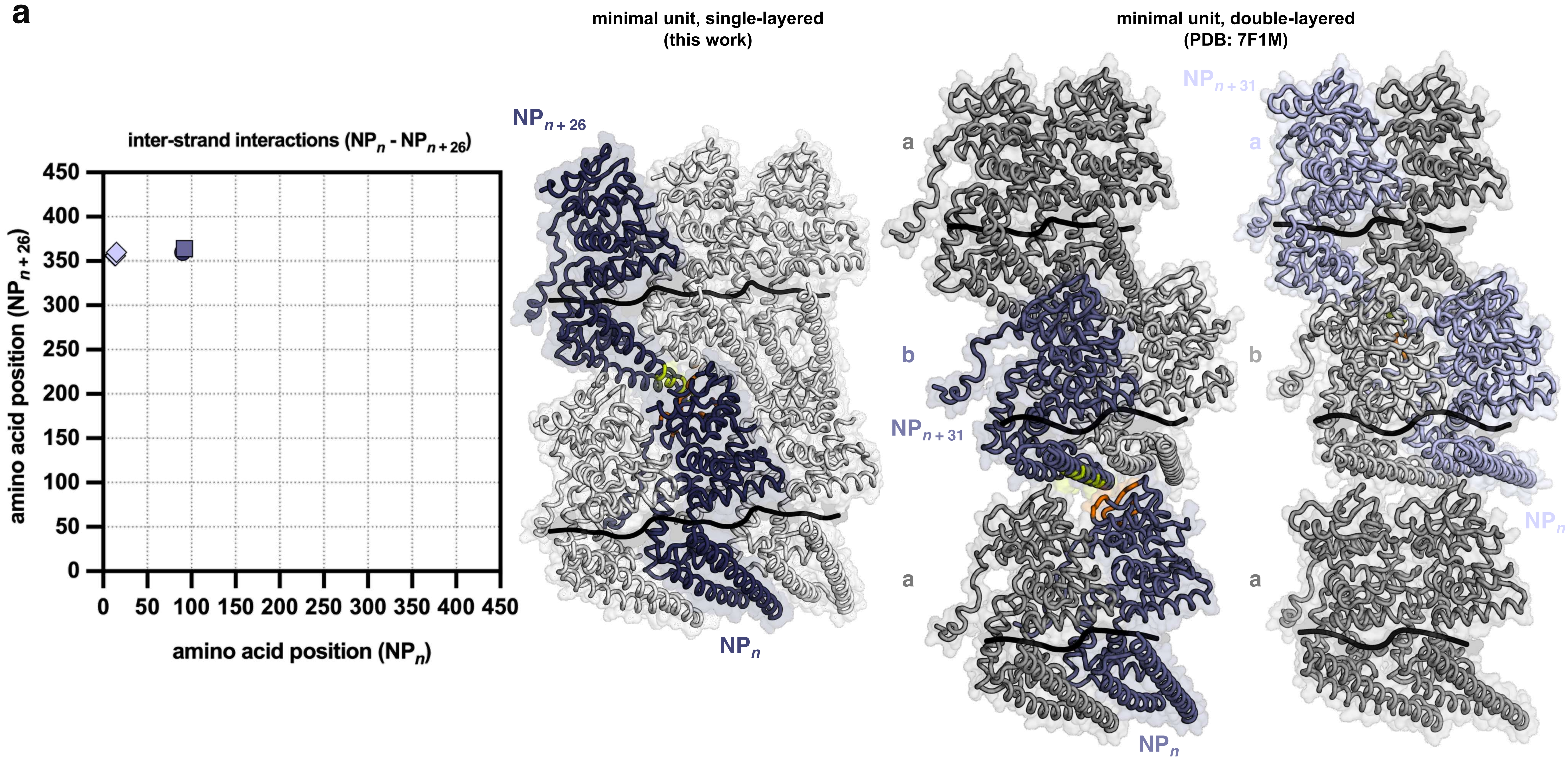

(6.0 Å distance cutoff)	number of interactions	residues from protomer NP$_n$	residues from protomer NP$_{n+26\,(31)}$	interface area (Å^2)
● NP (single-layered)	1	1	1	15.2
■ NP-a (double layered)	1	1	1	47.7
◇ NP-b (double-layered)	3	3	2	15.1

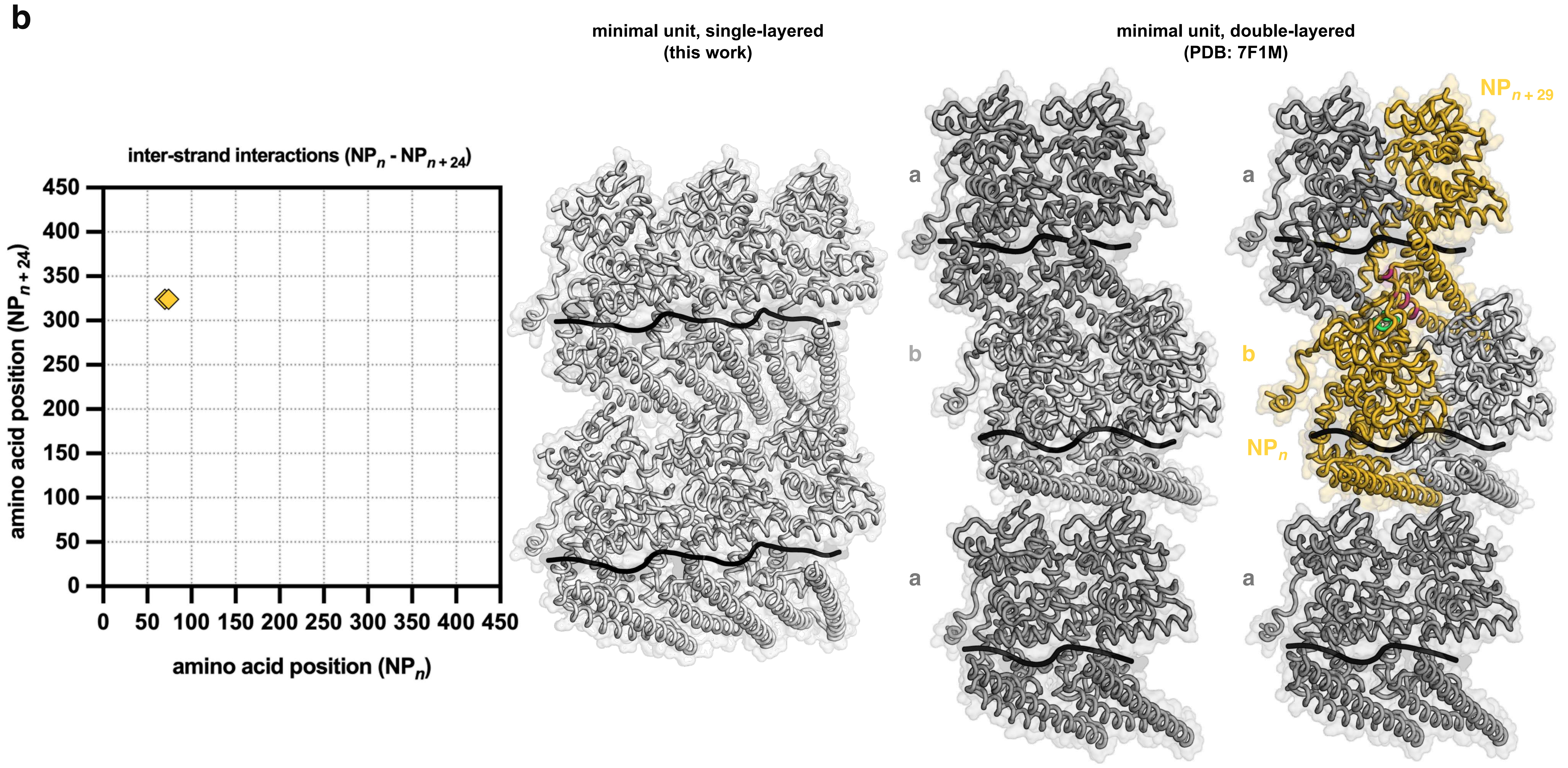

(6.0 Å distance cutoff)	number of interactions	residues from protomer NP$_n$	residues from protomer NP$_{n+24\,(29)}$	interface area (Å^2)
NP (single-layered)	0	0	0	0
NP-a (double layered)	0	0	0	0
◇ NP-b (double-layered)	2	2	1	55.1

Supplementary Fig. 12. Structural analysis of NP$_{core}$ - NP$_{core}$ interactions in the two conformations of the MARV RNP complex. **a**, amino acid residue contacts map (left panel), ribbon/isosurface representation (right panel) and quantitative comparison (lower panel) of the inter-strand interactions between any given NP$_n$ and the neighbor protomer in the subsequent position of the subsequent strand, NP$_{n+26}$ (this work) or NP$_{n+31}$ (PDB: 7F1M). **b**, amino acid residue contacts map (left panel), ribbon/isosurface representation (right panel) and quantitative comparison (lower panel) of the inter-strand interactions between any given NP$_n$ and the neighbor protomer in the precedent position of the subsequent strand, NP$_{n+24}$ (this work) or NP$_{n+29}$ (PDB: 7F1M). For any arbitrary protomer NP in the single- and NP-a and NP-b in the double-layer MARV RNP complex, respectively, the number of interactions, involved residues per protomer and extension of the interface area are indicated (circle, single-layered RNP NP; square, double-layered RNP NP-a; rhombus, double-layered RNP NP-b).

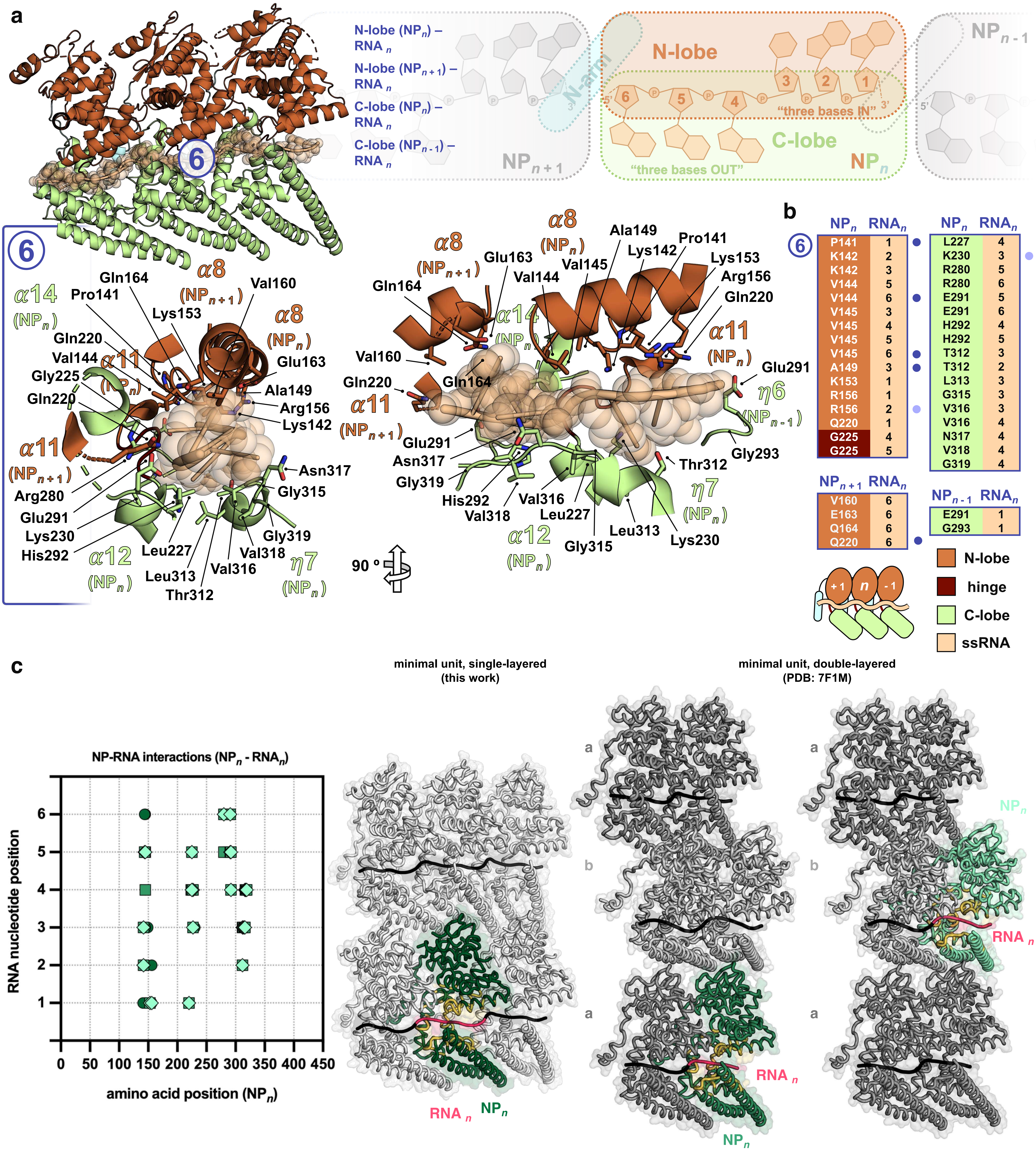

(4.0 Å distance cutoff)	number of interactions	residues from protomer NP$_n$	residues from protomer RNA $_n$	interface area (Å^2)
● NP (single-layered)	33	21	6	801.9
■ NP-a (double layered)	26	18	6	751.1
◇ NP-b (double-layered)	25	18	6	746.1

Supplementary Fig. 13. Structural details of single-layered MARV RNP complex (RNA binding) and analysis of NP$_{core}$ - RNA interactions in two conformations of the MARV RNP complex. a, ribbon representation overview of a trimeric minimal unit (top left), cartoon of ssRNA "three bases-in, three bases-out" arrangement (top right) and close-up (insets) showing the interactions between any arbitrary RNA$_n$ and the N-lobe and C-lobe of NP$_n$, NP$_{n+1}$ and NP$_{n-1}$ protomers (this work). **b**, summary of interacting residues (within 4 Å distance) of MARV NP$_{core}$ with ssRNA; residue-nucleotide couples marked with a circle are unique (within 4 Å distance) of the MARV RNP complex in single-layer conformation (with R156 and K230 also present, at longer distance, in the double-layer one). **c**, amino acid and ribonucleotide residue contacts map (left panel), ribbon/isosurface representation (right panel) and quantitative comparison (lower panel) of the interactions between any given NP$_n$ protomer and its bound RNA$_n$ of NP (this work), or NP-a and NP-b (PDB: 7F1M) protomers. For any arbitrary protomer NP in the single-, and NP-a and NP-b in the double-layer MARV RNP complex, respectively, the number of interactions, involved residues per protomer and extension of the interface area are indicated (circle, single-layered RNP NP; square, double-layered RNP NP-a; rhombus, double-layered RNP NP-b).

a

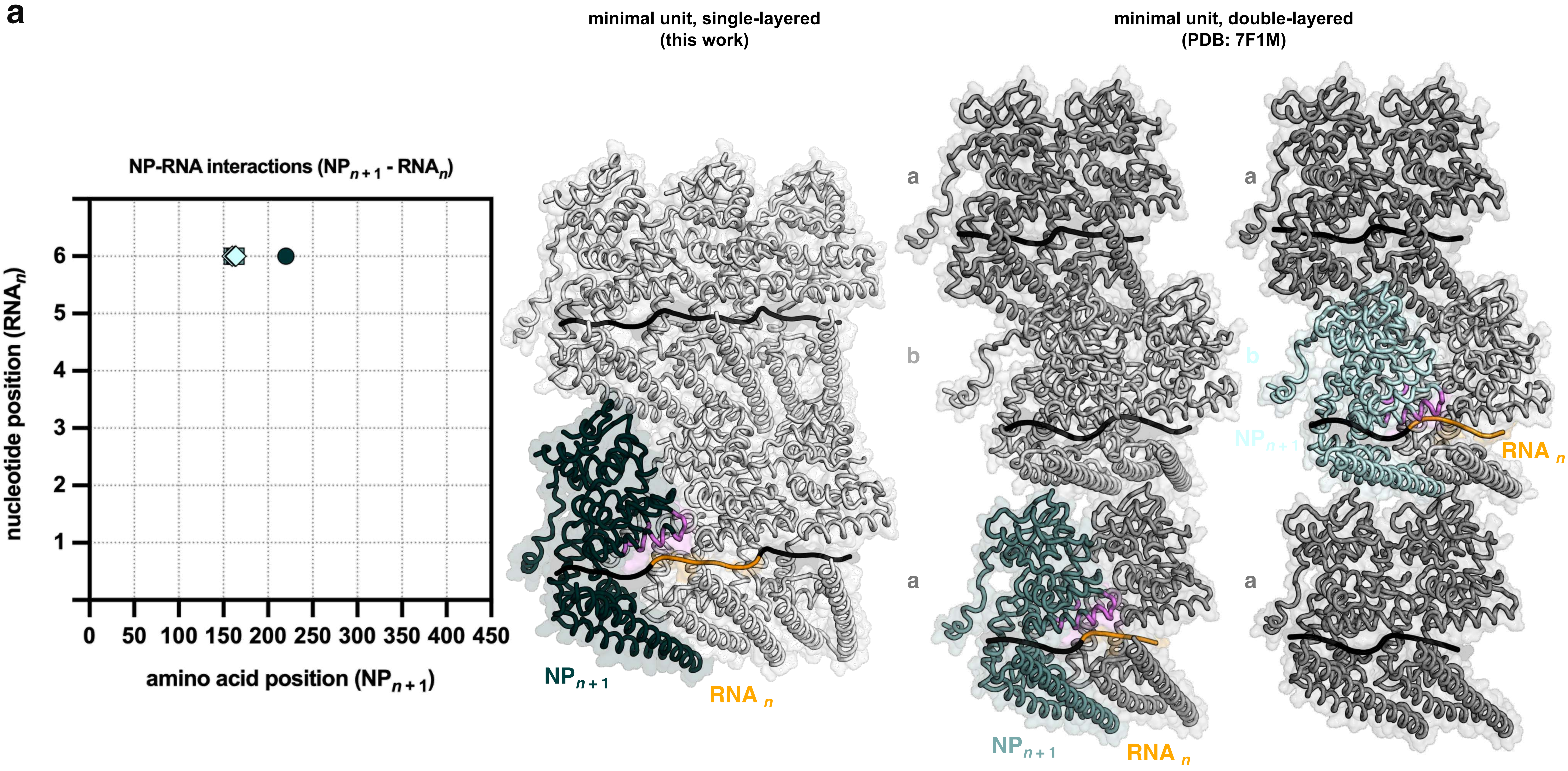

(4.0 Å distance cutoff)	number of interactions	residues from protomer NP$_{n+1}$	ribonucleotides from protomer RNA$_n$	interface area (Å^2)
NP (single-layered)	4	4	1	145.2
NP-a (double layered)	3	3	1	162.0
NP-b (double-layered)	3	3	1	132.3

b

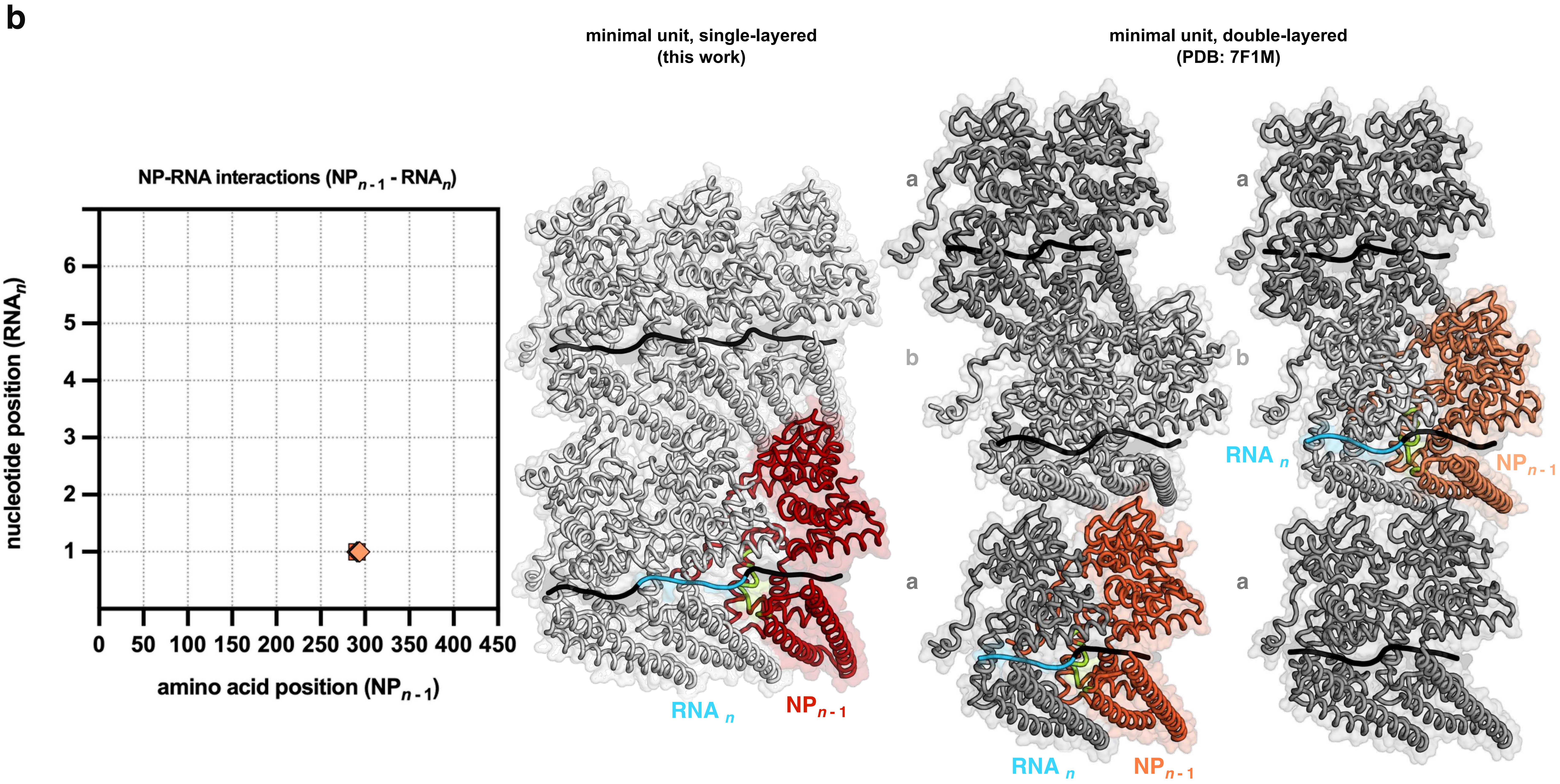

(4.0 Å distance cutoff)	number of interactions	residues from protomer NP$_{n-1}$	ribonucleotides from protomer RNA$_n$	interface area (Å^2)
NP (single-layered)	2	2	1	106.7
NP-a (double layered)	1	1	1	100.1
NP-b (double-layered)	3	3	1	91.2

Supplementary Fig. 14. Structural analysis of NP$_{core}$ - RNA interactions in the two conformations of the MARV RNP complex. **a**, amino acid and ribonucleotide residue contacts map (left panel), ribbon/isosurface representation (right panel) and quantitative comparison (lower panel) of the interactions between any given RNA$_n$ and its adjacent neighbor NP$_{n+1}$ protomer. **b**, amino acid and ribonucleotide residue contacts map (left panel), ribbon/isosurface representation (right panel) and quantitative comparison (lower panel) of the interactions between any given RNA$_n$ and its adjacent neighbor NP$_{n-1}$ protomer. For any arbitrary protomer NP (this work) in the single-, and NP-a and NP-b (PDB: 7F1M) in the double-layer MARV RNP complex, respectively, the number of interactions, involved residues per protomer and extension of the interface area are indicated (circle, single-layered RNP NP; square, double-layered RNP NP-a; rhombus, double-layered RNP NP-b).

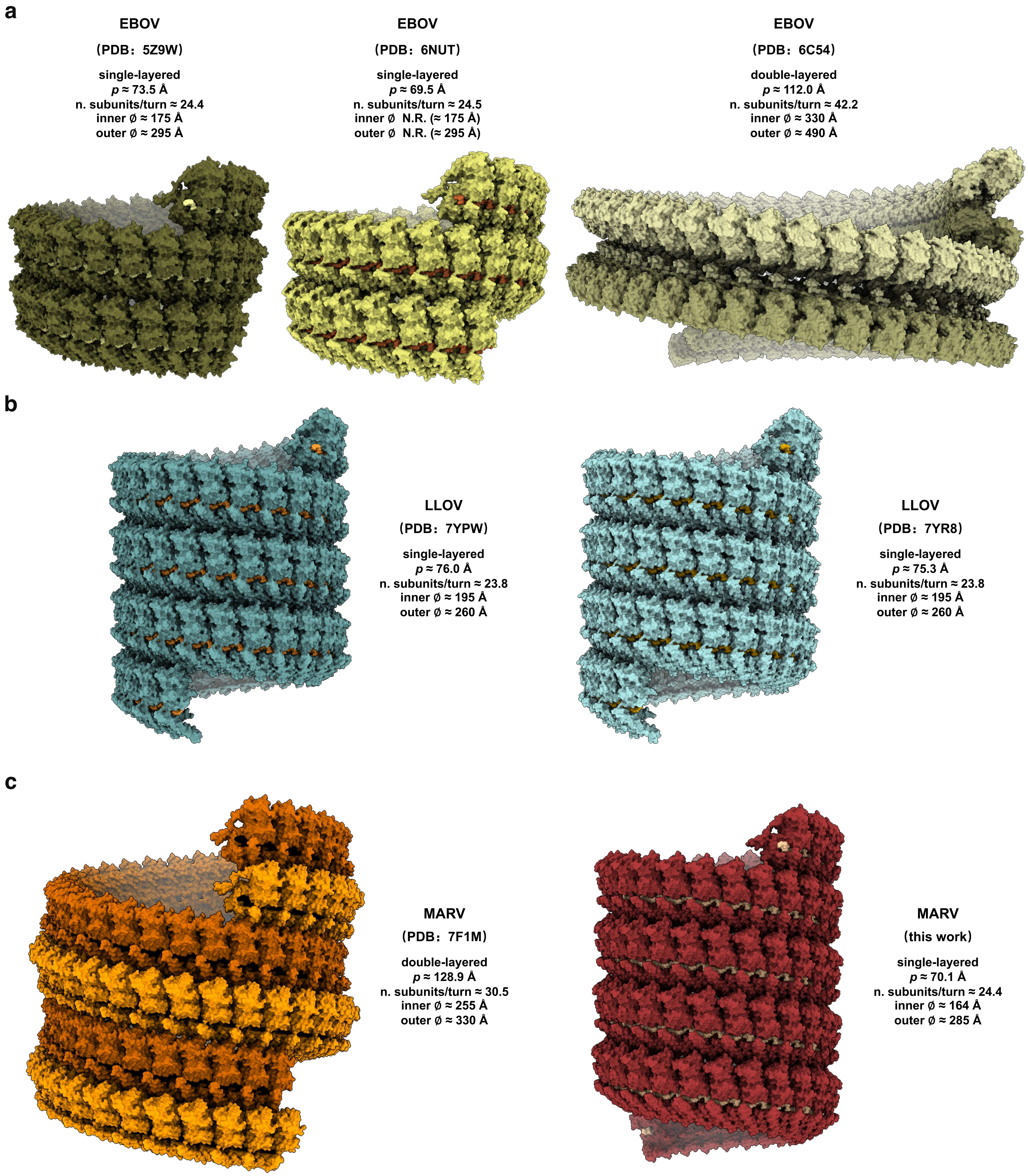

Supplementary Fig. 15. Comparison between single- and double-layered RNP complexes among filoviruses. Atomic models of cryo-EM RNP complexes from the *Filoviridae* family, including members of: **a**, the *Orthoebolavirus* genus, i.e., EBOV in single- (left, PDB: 5Z9W; middle, PDB: 6NUT) and double-layer (right, PDB: 6C54) conformation; **b**, the *Cuevavirus* genus, i.e., LLOV in single-layer conformation of full-length NP (left, PDB: 7YPW) and NP_{core} (right, PDB: 7YR8); **c**, the *Orthomarburgvirus* genus, i.e., MARV in double- (left, PDB: 7F1M) single-layer (this work, right) and conformation, all displayed as isosurface representations in side view orientation. For each atomic model the major helical parameters are indicated (*p*, pitch; ∅, diameter; N.R., not reported).
