# Supplementary material for: Cryo-EM structure of single-layered nucleoprotein-RNA complex from Marburg virus
Source: Nat Commun. 2024 Nov 27;15:10307. doi: 10.1038/s41467-024-54431-7 (PMC11603049; doi:10.1038/s41467-024-54431-7)
Supplement: Supplementary file 2 — Reporting Summary [file 41467_2024_54431_MOESM2_ESM.pdf]

nature portfolio

Corresponding author(s): Luca Zinzula, Wolfgang Baumeister

Last updated by author(s): 2024-31-10

Reporting Summary

Nature Portfolio wishes to improve the reproducibility of the work that we publish. This form provides structure for consistency and transparency in reporting. For further information on Nature Portfolio policies, see our Editorial Policies and the Editorial Policy Checklist.

Statistics

For all statistical analyses, confirm that the following items are present in the figure legend, table legend, main text, or Methods section.

n/a	Confirmed	
☐	☒	The exact sample size (*n*) for each experimental group/condition, given as a discrete number and unit of measurement
☐	☒	A statement on whether measurements were taken from distinct samples or whether the same sample was measured repeatedly
☒	☐	The statistical test(s) used AND whether they are one- or two-sided<br>*Only common tests should be described solely by name; describe more complex techniques in the Methods section.*
☒	☐	A description of all covariates tested
☒	☐	A description of any assumptions or corrections, such as tests of normality and adjustment for multiple comparisons
☒	☐	A full description of the statistical parameters including central tendency (e.g. means) or other basic estimates (e.g. regression coefficient) AND variation (e.g. standard deviation) or associated estimates of uncertainty (e.g. confidence intervals)
☒	☐	For null hypothesis testing, the test statistic (e.g. *F*, *t*, *r*) with confidence intervals, effect sizes, degrees of freedom and *P* value noted<br>*Give P values as exact values whenever suitable.*
☒	☐	For Bayesian analysis, information on the choice of priors and Markov chain Monte Carlo settings
☒	☐	For hierarchical and complex designs, identification of the appropriate level for tests and full reporting of outcomes
☒	☐	Estimates of effect sizes (e.g. Cohen's *d*, Pearson's *r*), indicating how they were calculated

Our web collection on statistics for biologists contains articles on many of the points above.

Software and code

Policy information about availability of computer code

Data collection	Negative stain EM and cryo-EM data collection was performed manually using the open-source software Serial EM (ref. 30), and automatically using the commercial software EPU (ThermoFisher Scientific), respectively.
Data analysis	Negative stain micrographs were visually inspected by using the open-source software Fiji (ref.31). Cryo-EM micrograph datasets was processed by using open-source software RELION MotionCor implementation (ref. 32), CTFFIND4 (ref. 33), RELION 4.0 (ref. 34), crYOLO (ref. 35), DeepEMhancer (ref. 36), TOM-toolbox (ref. 37), and the commercial software MATLAB (MathWorks). Atomic model building and refinement was performed by using the open-source software AlphaFold2 (ref. 38), Coot (ref. 39), Phenix (ref. 40), Lsqkab (ref. 41). Molecular graphics were produced by using open-source software Chimera (ref. 42), ChimeraX (ref. 43) and commercial software Prism (GraphPad) and PyMOL (Schrödinger). Structural bioinformatics analysis was performed by using open-source tools TM-align (ref. 44), MatchMaker and Match-Align (ref. 45), PDB2PQR (ref. 46), APBS (ref. 47), ESPript (ref. 48), Clustal Omega (ref. 49), ConSurf (ref. 50), SOCKET2 (ref. 52) and COCOMAPS (ref. 53).

For manuscripts utilizing custom algorithms or software that are central to the research but not yet described in published literature, software must be made available to editors and reviewers. We strongly encourage code deposition in a community repository (e.g. GitHub). See the Nature Portfolio guidelines for submitting code & software for further information.

Data

Policy information about availability of data

All manuscripts must include a data availability statement. This statement should provide the following information, where applicable:

- Accession codes, unique identifiers, or web links for publicly available datasets
- A description of any restrictions on data availability
- For clinical datasets or third party data, please ensure that the statement adheres to our policy

The atomic coordinates of single-layered MARV RNP complex structure generated in this work have been deposited in the PDB with the accession code 9FVD; the corresponding cryo-EM density maps have been deposited in the EMDB with the accession codes EMD-50803 and EMD-50804, for the helical assembly and the RNA-bound trimeric unit, respectively. For comparative analysis, atomic coordinates from other works including those with accession codes 5F5M, 5F5O, 5XSQ, 5Z9W, 6C54, 6NUT, 7F1M, 7YPW and 7YR8, and cryo-EM and cryo-ET density maps including EMD-31420 and EMD-3875, were obtained from PDB and EMDB, respectively. Any other raw data related to this work are available from the corresponding authors upon reasonable request. Source data are provided with this paper. A GitHub repository containing the code used in the 2D analysis of helix diameter and power spectra is available at https://github.com/FlorianBeckOle/2dClassAnalysis.git.

Research involving human participants, their data, or biological material

Policy information about studies with human participants or human data. See also policy information about sex, gender (identity/presentation), and sexual orientation and race, ethnicity and racism.

Reporting on sex and gender	Not applicable
Reporting on race, ethnicity, or other socially relevant groupings	Not applicable
Population characteristics	Not applicable
Recruitment	Not applicable
Ethics oversight	Not applicable

Note that full information on the approval of the study protocol must also be provided in the manuscript.

Field-specific reporting

Please select the one below that is the best fit for your research. If you are not sure, read the appropriate sections before making your selection.

[x] Life sciences   [ ] Behavioural & social sciences   [ ] Ecological, evolutionary & environmental sciences

For a reference copy of the document with all sections, see nature.com/documents/nr-reporting-summary-flat.pdf

Life sciences study design

All studies must disclose on these points even when the disclosure is negative.

Sample size	No calculations were performed to design a predetermined sample size for this study. The number of particles (n=67,385) and the number of micrographs (n=5,518) in the cryo-EM dataset were the result of the density of particles in the protein sample applied to the electron microscopy grid and of the available microscope operating time, respectively. Both numbers were validated as sufficient because they could lead to the obtainment of a high-resolution 3D reconstruction of the biological specimen.
Data exclusions	No collected data were excluded a priori before the analysis. Nevertheless, as per characteristics of the single particle analysis method, micrographs, image segments and particles misaligned or not fulfilling cross-correlation scores were iteratively classified during the image processing workflow and excluded from subsequent processing steps.
Replication	No replicates for cryo-EM dataset collection and 3D reconstruction of the specimen were performed. Expression, purification and EM sample preparation were repeated several times for protocol optimization and until optimal protein yield, purity and homogeneity was achieved. All replication attempts were successful.
Randomization	Randomization in this study was not required because experiments did not include allocation into experimental groups. Nevertheless, as per characteristics of the single particle analysis method, during the image processing workflow particles were randomly and automatically subdivided into two sub-datasets to independently obtain two EM density half-maps and calculate the gold-standard Fourier Shell correlation score for the resolution estimation.
Blinding	Blinding in this study was not required because experiments did not include allocation into experimental groups. Nevertheless, as per characteristics of the single particle analysis method, reference-free 2D classification of particles and featureless cylinder-template based reconstruction of the 3D initial model were performed to avoid bias.

Reporting for specific materials, systems and methods

We require information from authors about some types of materials, experimental systems and methods used in many studies. Here, indicate whether each material, system or method listed is relevant to your study. If you are not sure if a list item applies to your research, read the appropriate section before selecting a response.

Materials & experimental systems

n/a	Involved in the study
☒	☐ Antibodies
☒	☐ Eukaryotic cell lines
☒	☐ Palaeontology and archaeology
☒	☐ Animals and other organisms
☒	☐ Clinical data
☒	☐ Dual use research of concern
☒	☐ Plants

Methods

n/a	Involved in the study
☒	☐ ChIP-seq
☒	☐ Flow cytometry
☒	☐ MRI-based neuroimaging

Plants

Seed stocks	Not applicable
Novel plant genotypes	Not applicable
Authentication	Not applicable
